# Combinations of SNPs Related to Signal Transduction in Bipolar Disorder

**DOI:** 10.1371/journal.pone.0023812

**Published:** 2011-08-29

**Authors:** Pernille Koefoed, Ole A. Andreassen, Bente Bennike, Henrik Dam, Srdjan Djurovic, Thomas Hansen, Martin Balslev Jorgensen, Lars Vedel Kessing, Ingrid Melle, Gert Lykke Møller, Ole Mors, Thomas Werge, Erling Mellerup

**Affiliations:** 1 Laboratory of Neuropsychiatry, Department of Neuroscience and Pharmacology, University of Copenhagen, Copenhagen, Denmark; 2 Psychiatric Centre Copenhagen, Department O, Copenhagen University Hospital, Rigshospitalet, Copenhagen, Denmark; 3 Department of Psychiatry, Oslo University Hospital and Institute of Psychiatry, University of Oslo, Oslo, Norway; 4 Department of Medical Genetics, Oslo University Hospital and Institute of Psychiatry, University of Oslo, Oslo, Norway; 5 Department of Biological Psychiatry, Mental Health Centre Sct. Hans, Copenhagen University Hospital, Roskilde, Denmark; 6 Centre for Psychiatric Research, Aarhus University Hospital, Risskov, Denmark; 7 Genokey ApS, ScionDTU, Technical University of Denmark, Hoersholm, Denmark; Leiden University Medical Center, The Netherlands

## Abstract

Any given single nucleotide polymorphism (SNP) in a genome may have little or no functional impact. A biologically significant effect may possibly emerge only when a number of key SNP-related genotypes occur together in a single organism. Thus, in analysis of many SNPs in association studies of complex diseases, it may be useful to look at combinations of genotypes. Genes related to signal transmission, e.g., ion channel genes, may be of interest in this respect in the context of bipolar disorder. In the present study, we analysed 803 SNPs in 55 genes related to aspects of signal transmission and calculated all combinations of three genotypes from the 3×803 SNP genotypes for 1355 controls and 607 patients with bipolar disorder. Four clusters of patient-specific combinations were identified. Permutation tests indicated that some of these combinations might be related to bipolar disorder. The WTCCC bipolar dataset were use for replication, 469 of the 803 SNP were present in the WTCCC dataset either directly (n = 132) or by imputation (n = 337) covering 51 of our selected genes. We found three clusters of patient-specific 3×SNP combinations in the WTCCC dataset. Different SNPs were involved in the clusters in the two datasets. The present analyses of the combinations of SNP genotypes support a role for both genetic heterogeneity and interactions in the genetic architecture of bipolar disorder.

## Introduction

Bipolar disorder (BD) is a severe psychiatric disease generally characterized by repeated manic and depressive episodes. The time of onset varies, but it usually begins between the ages of 15 and 25 years, affecting men and women equally with a lifetime prevalence of 1.5% [1]. Family, twin, and adoption studies have shown that genetic factors contribute to BD, but the involved genes have not yet been identified. This may partly be explained by a genetic architecture characterized by both genetic heterogeneity (at the population level) and polygenic interactions (at the level of the individual) [2]–[5]. Locus heterogeneity in a disease may occur when dysfunctions in different mechanisms each produce the symptoms characterising the phenotype.

In the search for disease-associated genes, high-throughput genotyping methods have allowed analysis of a large number of single nucleotide polymorphisms (SNPs), but most of these studies have used single-locus analysis strategies. Yet, many common diseases have complex aetiologies that may involve combinations of SNPs from different genes and, possibly, different combinations within the population of affected individuals. The growing interest in interactions and their contribution to risk for complex diseases has resulted in a search for methods of calculating disease-related interactions of two or more SNPs [6]. Most of these methods are theoretical, and many concentrate on finding combinations of two SNPs [7]–[10]. Only a few studies have looked at combinations of more SNP genotypes; for example, Xie et al. [11], in a study of oesophageal cancer, analysed SNP genotypes from genes related to DNA repair mechanisms and liver function and identified combinations of genotypes relevant to the disease. In the quest for alternative approaches in the search for interacting SNPs, interest has grown in pathways with an enrichment of associated signals [12]–[15]. These methods are robust to the detection of enrichment that derives from genetic heterogeneity at the population level or from gene- or protein-interactions at the individual level.

In BD, hyperactivity is the main symptom of the manic phase, and this clinical phenotype may reflect altered activity at the cellular or molecular level that leads to faster signal transmission in the brain. Numerous mechanisms are involved in signal transmission, suggesting that the mania phenotype may result from many different dysfunctions in mechanisms relevant to signal transmission velocity in the nervous system; this concept is in agreement with the genetic heterogeneity of BD [2], [3]. We previously suggested that propagation of nerve impulses may be faster in mania than in a normal affective state [16], which is in agreement with a number of similar hypotheses proposing mania as a disorder of ionic conductance of nerve cell membranes [17], nerve cell excitability [18], [19], action potential firing [20], neuronal hypersynchronization [21], changes in sodium pump number [22], membrane abnormalities [23], and cortical instability [24]. Furthermore, the suggestion that nervous system hyperactivity may play a role in BD is in accordance with the fact that several drugs used to treat epilepsy are effective in the treatment of mania.

We have investigated genes (1) related to the action potential, where we focused on genes related to the architecture of the sodium channels in the nodes of ranvier and CNS myelination [19], [25]–[29]. Among these genes, we have selected those (2) that occur in loci considered to be of interest with respect to BD, as indicated by linkage analyses [30], [31]; (3) that encode proteins that are targets for mood-stabilizing drugs [29], [31], [32]; or (4) that previous studies have shown are associated with BD [19], [26], [29], [31]. Many genes in this group are related to ion channels, an observation that agrees with the finding of Askland et al. [12] that variation in ion channel genes may contribute to susceptibility to BD.

In the present study, we analysed 803 SNPs in 55 genes related to aspects of signal transmission and calculated all combinations of three genotypes (the normal homozygote, the heterozygote, and the variant homozygote, respectively) from the 3×803 SNP genotypes. We likewise calculated all combinations with three SNP genotypes from the genome wide association study (GWAS) performed by the Wellcome Trust Case Control Consortium (WTCCC) [33] using SNPs analysed in both datasets. We found 132 SNPs that were genotyped in both studies, by imputation we obtained additional 337 SNPs with a good quality, thus 469 SNPs were among our 803 SNPs and available for validation.

## Results

### Calculation of single SNP association

803 SNPs in 55 genes (see Table 1) were analysed and a Chi-square test (or Fishers exact test when appropriate) was performed for each SNP. The genotype distribution was significantly different (p<0.05) between control persons and patients for 86 SNPs (see Table S1), but none remained significant after a Bonferroni correction.

**Table 1:** Selected genes, function, and number of SNPs.

Gene	Location	Name and/or Function	SNP	Ref.^a^
*ANK3*	10q21	Role for structure and function of nodes of Ranvier	84	25,28,29
*AQP4*	18q11.2–q12.1	Regulator of vasopression secretion	12	30,31
*ATP1A2*	1q21–q23	Na+/K+ ATPase alpha-2 subunit	10	30,32
*ATP1A3*	19q13.31	Na+/K+ ATPase alpha-3 subunit	3	29,30
*AVPR1B*	1q32	Arginine vasopressin receptor 1B	3	30,42
*BACE1*	11q23.2–q23.3	Regulation of the voltage dependent Na-channels.	10	30,40
*BDNF*	11p13	Involved in neuroplasticity and stress response	6	29–31
*CACNG2*	22q13.1	Neuronal calcium channel gamma subunit, stabilize the channel in an inactive state	34	30,43
*CAMKK2*	12q24.2	Involved in activation of CREB1	10	30–32
*CLDN11*	3q26.2–q26.3	Role in myelinisation	3	26,30,31
*CNTN1*	12q11–q12	Cell adhesion molecule	30	25,32
*CNTN2*	1q32.1	Cell adhesion molecule	14	25,30
*CNTNAP1*	17q21	Contactin-associated protein, may be the signaling subunit of contactin	2	25,28
*CNTNAP2*	7q35–q36	Cluster voltage-gated potassium channels, localized at the juxtaparanodes	46	25,27,28,30
*CREB1*	2q34	Transcription factor	5	28,30,32
*DLG4*	17p13.1	Neuronal development, recruited into potassium channel clusters	6	27,30,32
*ERBB4*	2q33.3–q34	Neuregulin-1 receptor, involved in mitogenesis and differentiation	6	30,41
*GSK3B*	3q13.3	Neuronal cell development (Related to lithium respons)	4	29,31
*IMPA2*	18p11.2	Inositol monophosphatase (Related to lithium respons)	16	29,30
*KCNA1*	12p13.32	Voltage-gated delayed potassium channel	2	25,27,30
*KCNA2*	1p13	Voltage-gated delayed potassium channel, delayed rectifier class	2	25,27,30
*KCNC1*	11p15	Mediates the voltage-dependent potassium ion permeability of excitable membranes	9	30,38
*KCNC2*	12q14.1	Mediates the voltage-dependent potassium ion permeability of excitable membranes	26	30,32
*KCNC3*	19q13.3–q13.4	Mediates the voltage-dependent potassium ion permeability of excitable membranes	5	30,38
*KCNN3*	1q21.3	Potassium conductance Ca-activited channel, regulate neuronal excitability	29	30
*KCNQ2*	20q13.3	Voltage-gated potassium channel plays a role in the regulation of neuronal excitability	12	25,29,30
*KCNQ3*	8q24	Voltage-gated potassium channel plays a role in the regulation of neuronal excitability	30	25,30
*MAG*	19q13.1	Central role i myelinisation, involved in myelin-neuron cell-cell interactions	8	26,30
*MAP2*	2q34–q35	Microtubule-associated protein, involved in neurogenesis	16	30,31
*MBP*	18q23	Major constituent of the myelin sheath of oligodendrocytes in the nervous system	23	26,30
*MCHR1*	22q13.2	Inhibit cAMP accumulation stimulate intracellular Ca-flux	3	26,29,30
*MCTP2*	15q26.2	Intercellular signal transduction	3	30
*MOG*	6p22.1	Involved in completion and maintenance of the myelin sheath and in cell-cell communication	7	26
*NCAM1*	11q23.1	Neural cell adhesion molecule 1	34	25,28–30
*NFASC*	1q32.1	Cell adhesion; organization of the axon initial segment (AIS) and nodes of Ranvier	31	25,28,30
*NRCAM*	7q31.1–q31.2	Ankyrin-binding protein is involved in neuron-neuron adhesion	37	25,29,30
*NRG1*	8p12	Associated with ERBB receptors	38	26,29,30,41
*NTRK1*	1q21–q22	Neurotrophic tyrosine kinase, receptor, type 1	1	30,39
*OLIG2*	21q22.11	Oligodendrocyte lineage transcription factor 2	3	26,30
*P2RX7*	12q24	Ligand-gated ion channel	19	29,30
*PDE4B*	1p31	Phosphodiesterase 4B, cAMP-specific	3	30,32
*PPP2R2C*	4p16.1	Protein phosphatase 2, regulatory subunit B, gamma isoform	27	29,30
*SCN1B*	19q13.1	Sodium channel beta subunit, propagation of nerveimpulses, binding to contactin	3	25,28,30
*SCN2A*	2q23–q24	Sodium channel alpha subunit, generation and propagation of action potentials in neurons	12	25,28,30
*SCN2B*	11q23	Sodium channel, voltage-gated, type II, beta	5	25,28,30
*SCN4B*	11q23.3	Sodium channel, voltage-gated, type IV, beta	6	25,28,30
*SCN5A*	3p21	Sodium channel, voltage-gated, type V, alpha subunit	5	25,28,30
*SCN8A*	12q13	Sodium channel, voltage gated, type VIII, alpha subunit, associated with ANK3	15	25,28,30
*SLC12A6*	15q13–q15	Electroneutral potassium-chloride cotransporter 3	10	29,30
*SPTBN4*	19q13.13	Involved in location of specific membrane proteins in polarized regions of neurons	15	25,28,30
*TBR1*	2q24	Transcription factor, critical for early cortical development	3	30,31
*TNC*	9q33	Regulation of Na channels. Interaction with CNTN1	28	25,30
*TNR*	1q24	Extracellular matix protein expressed primarily in the central nervous system	16	25,30
*TRPM2*	21q22.3	Transient receptor potential cation channel, subfamily M, member 2	9	29,30
*YWHAH*	22q12.3	Tyrosine 3-monooxygenase/tryptophan 5-monooxygenase activation protein, eta polypeptide	4	29,30

a )As of September 2007, when our SNP selection ended prior to analysis.

### Combination of two SNPs

Combinations of two SNPs from 803 SNPs results theoretically in 803!/2!(803−2)!×9 = 2,898,027 combinations, whereas the actual number in the material was 2,770,033 combinations. Chi-square test for each of these combinations was not performed, but 1000 permutation tests showed that combinations found exclusively in the patients (161,070 combinations) could be random.

### Combinations with three SNPs

Combinations of three SNP genotypes from the 803 SNPs results theoretically in 803!/3!(803−3)!×3^3^ = 2,321,319,627 combinations of three-SNP genotypes (in the following to be termed 3-combinations) (Table 2). The actual number of 3-combinations of genotypes found in our material (1962 individuals) was 1,985,613,130. Most, namely 1,719,002,329 of these 3-combinations (87%) were common for both control persons and patients, whereas 208,699,590 3-combinations were found in control persons only, and 57,911,211 3-combinations were found in patients only (Table 2). The number of patient-specific 3-combinations shared by several patients decreases as the number of patients in a group increases (Table 2). When the number of patients was nine or more, only 1181 3-combinations of the 57,911,211 patient-specific 3-combinations remained. In order to see if the subgroup of 1181 3-combinations may be of importance for BD, 1000 permutation tests of the complete material were calculated, and it was found that 1181 3-combinations shared by nine or more patients might be a random finding (found 113 times; p = 0.11).

**Table 2:** Number of combinations of three SNP genotypes (3-combinations) found in 1355 control persons and 607 bipolar patients.

	Number of 3-combinations
Theoretical number with 803 SNPs	2,321,319,627
Found in the present material of 803 SNPs	1,985,613,130
Common for control persons and patients	1,719,002,329
Found in the control persons only	208,699,590
Found in the patients only	57,911,211
Found in single patients only and no control person	45,285,770
Common for 2 patients and no control person	9,557,540
Common for 3 patients and no control person	2,277,107
Common for 4 patients and no control person	578,259
Common for 5 patients and no control person	156,343
Common for 6 patients and no control person	41,019
Common for 7 patients and no control person	10,990
Common for 8 patients and no control person	3,002
Common for 9 patients and no control person	826
Common for 10 patients and no control person	261
Common for 11 patients and no control person	70
Common for 12 patients and no control person	22
Common for 13 patients and no control person	2
Common for 9 or more patients and no control person	1,181

**Figure 1:**
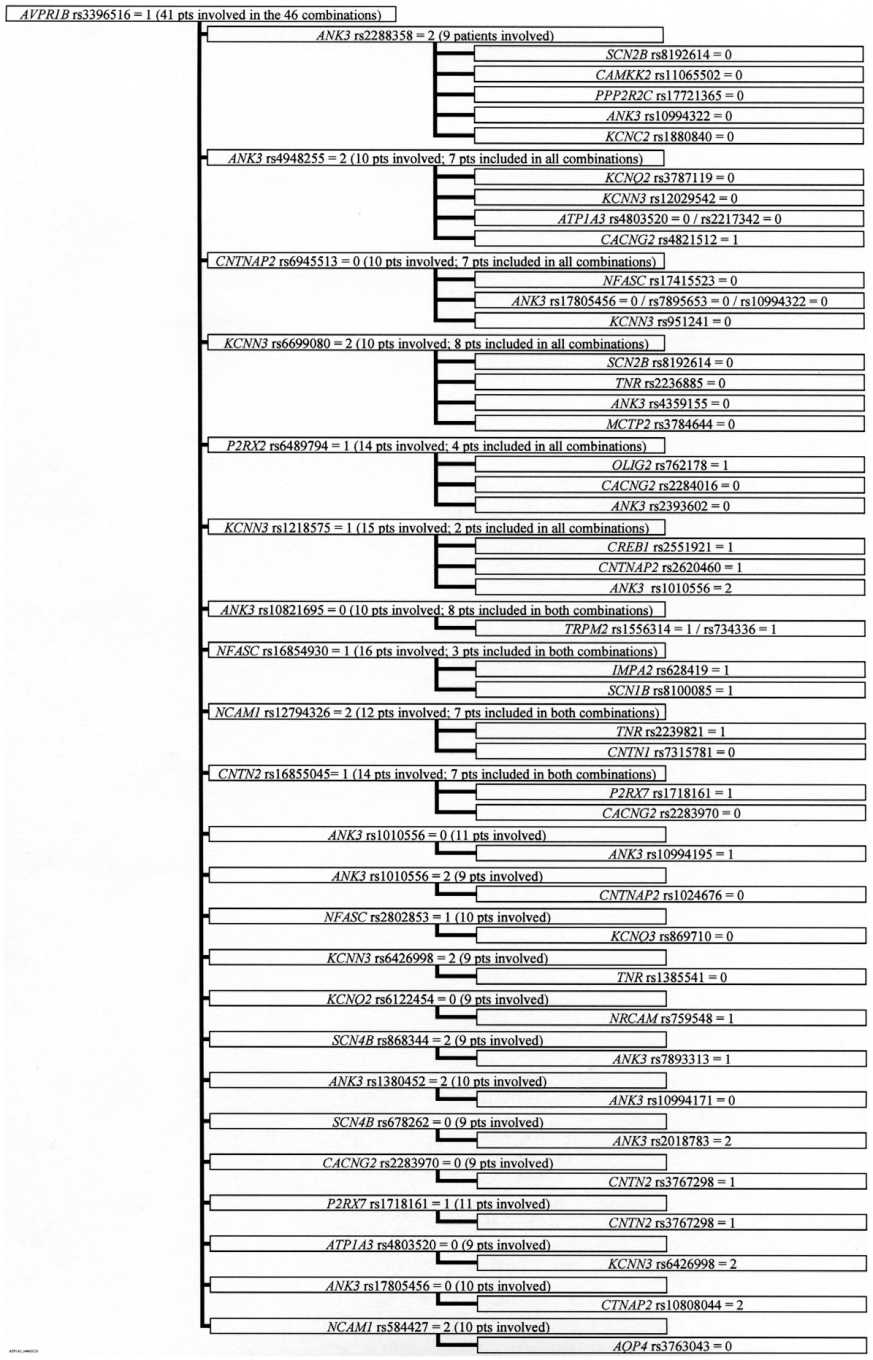
Cluster defined by *AVPR1B* rs33976516 = 1^a^. a) 0: Normal homozygote; 1: Heterozygote; 2: Variant homozygote.

Among the 1181 3-combinations, many genotypes were part of one or two 3-combinations and only a few genotypes occurred in more than ten 3-combinations. However, we observed that four genotypes (*AVPR1B*_rs33976516 = 1 (one indicate heterozygocity), *KCNN3*_rs884664 = 2 (two indicate homozygosity for the minor allele), *CACNG2*_rs2179871 = 2, *KCNQ3*_rs2469515 = 2) occurred in 46, 45, 49, and 32 3-combinations, respectively (Figure 1–4). The genotypes were all among the 86 SNPs showing a significantly different distribution between control persons and patients (nominal p-values were 0.013, 0.010, 0.023, and 0.017, respectively). Futhermore; these four observed subgroups of 3-combinations contained all the patients in the material (41, 48, 41, and 37 patients, respectively) having the four genotypes (Figure 1–4). Such subgroups characterised by relatively many 3-combinations sharing a defining genotype and containing all the patients having this genotype are called clusters. In 1000 permutation tests, at least one cluster of this type (with at least 37 pseudo-patients) were seen 42 times (p = 0.042), two such clusters were seen 3 times (p = 0.003), and at least three or more clusters of this type did not occur once (p<0.001). Relatively little overlap between the patients in the clusters was observed, as only eleven of the patients were members of two clusters, and no patient was a member of three or four clusters. A total of 156 patients were involved in the four clusters.

**Figure 2 pone-0023812-g002:**
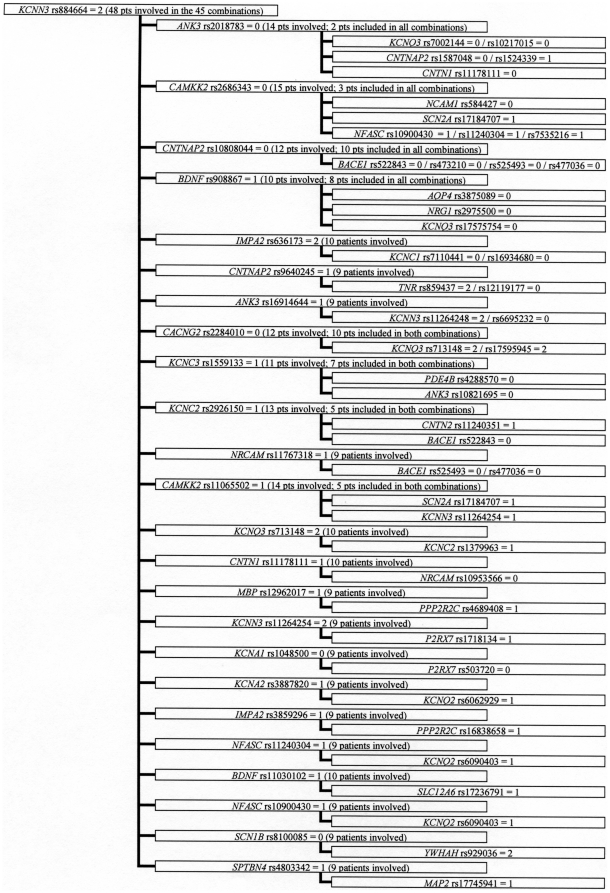
Cluster defined by *KCNN3* rs884664 = 2^a^. a) 0: Normal homozygote; 1: Heterozygote; 2: Variant homozygote.

**Figure 3 pone-0023812-g003:**
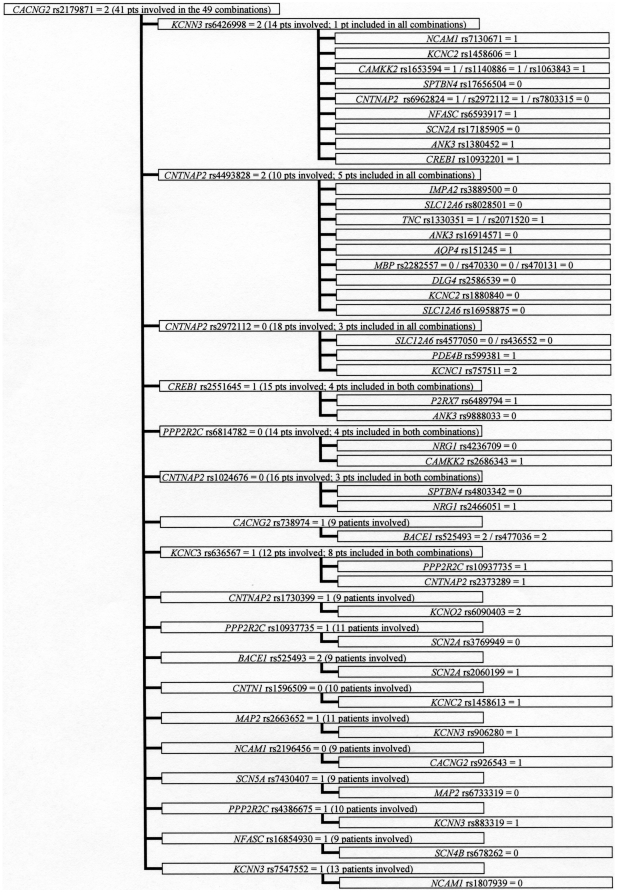
Cluster defined by *CACNG2* rs2179871 = 2^a^. a) 0: Normal homozygote; 1: Heterozygote; 2: Variant homozygote.

**Figure 4:**
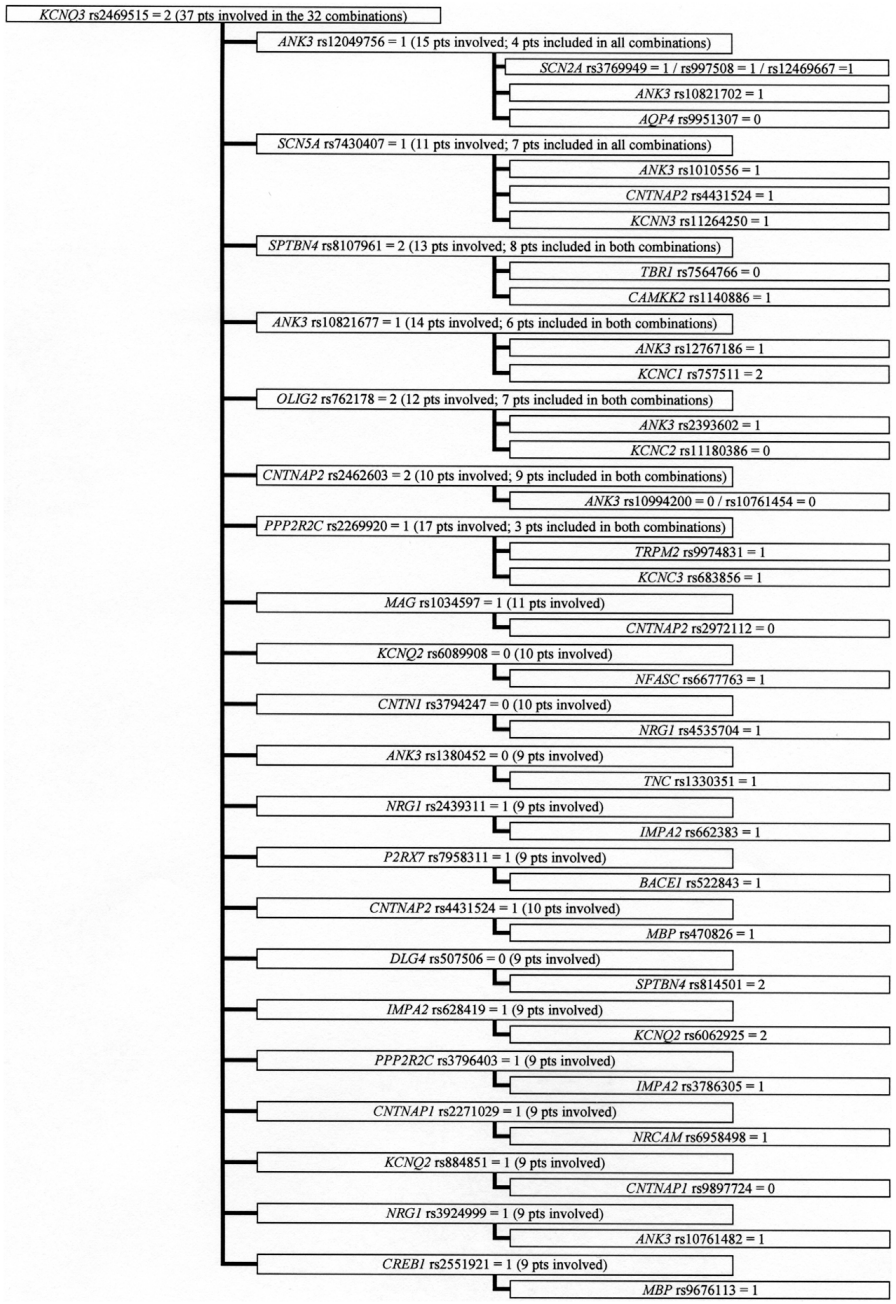
Cluster defined by *KCNQ3* rs2469515 = 2^a^. a) 0: Normal homozygote; 1: Heterozygote; 2: Variant homozygote.

All patients in a cluster shared the defining genotype, but nested within the four clusters, eleven smaller subgroups were observed. In these subgroups patients sharing the same defining genotype (e.g. *AVPR1B*_rs33976516 = 1) also shared a second genotype (e.g. *ANK3*_rs2288358) in combinations with four or more distinct third SNPs (shown in Figure 1–4 as the boxes in the third column). Considerable overlap between patients was found within these subgroups. In 1000 permutation tests only 49 subgroups of this type were found (p = 0.049). Additionally, within this type of subgroup, 12 even smaller subgroups were characterized by having the third genotype in the 3-combinations contributed by SNPs from the same gene (shown in Figure 1–4 as more SNP numbers in the same box). These 12 small subgroups together comprised 32 3-combinations. Only 19 3-combinations of this type were found in 1000 permutation tests (p = 0.019).

### WTCCC data [33]

Of our 803 SNPs 132 were genotyped in the WTCCC bipolar disorder dataset [33]. In order to obtain more SNPs, we performed an imputation using impute2 on the chromosome regions surrounding our selected genes. We could impute 651 SNPs of our 803 SNPs, but after removing SNPs with a low quality we ended up with additional 337 SNPs, leaving 469 SNPs in common between our material and the WTCCC.

Chi-square test (or Fishers exact test when appropriate) was performed for each SNP. The genotype distribution was significant different (p<0.05) between control persons and patients for 51 SNPs (see Table S1), but none remained significant after a Bonferroni correction. Five of the 51 SNPs were among the 86 significant SNPs in our material (see Table S1).

The theoretically number of 3-combinations from the 469 SNPs is 461,262,438, while the observed number was 287,931,183. Most, namely 247,477,254 were seen in both controls persons and patients, whereas 16,762,350 3-combinations were found in control persons only, and 23,691,570 3-combinations were found in patients only.

Using the above mention criteria for a cluster, three clusters were observed among the patient-specific 3-combinations. The defining genotypes were *NFASC*_rs12737855 = 2, *NFASC*_rs7519658 = 2 and *ANK3*_rs6479700 = 2, containing 124, 135 and 182 combinations, and 159, 150 and 142 patients, respectively (data not shown). All three genotypes were among the 51 significant SNPs. In 1000 permutation tests, at least one cluster of this type (with at least 142 pseudo-patients) were seen 49 times (p = 0.049), two such clusters were seen 3 times (p = 0.003), and at least three clusters of this type did not occur once (p<0.001).

Within the three clusters, we again observed smaller subgroups where patients also shared the second genotype in six or more combinations, and there were a considerable overlap between the patients involved in these subgroups. In 1000 permutation tests only 29 of such subgroups were found (p = 0.029).

There was a substantial overlap between the patients in the two clusters with the defining SNPs belonging to the *NFASC* gene (n = 134), while only a limited number of patients belonging to the third cluster (defined by *ANK3*_rs6479700) were present in the two other clusters (n = 10 and n = 8, respectively, where eight were part of all three clusters).

## Discussion

Complex diseases may be associated with combinations of SNPs. A number of methodological and theoretical studies have addressed this statistical and data-mining challenge [7]–[15], [34], but clinical investigations using combinations of several SNPs are rare [11]. The problem with combinations is the large numbers created, which is computationally demanding, especially when permutation tests are used as a statistical method. Thus in the present study, with 803 SNPs and combinations of no more than three SNP genotypes at a time, we found 1,985,613,130 3-combinations, close to the theoretical maximum of 2,321,319,627 3-combinations. Due to the relative low number of subjects and genetic factors as allele frequency and non-independence of SNPs located in the same gene region, we had expected to find a smaller number of 3-combinations.

In an attempt to identify 3-combinations related to BD, only 3-combinations found exclusively in the patient group were examined in more detail in this study, meaning that only combinations with 100% penetrance were examined. Among the 1,7 billion 3-combinations common for control persons and patients many may be associated with disease; these combinations will be analysed separately. In the 58 million 3-combinations found exclusively in the patients, 45 million were singularities (found in one person only), raising the possibility that they were random. Following this line of reasoning, the 1181 3-combinations, shared by nine or more patients and no control person, may be the most promising in relation to BD, but permutation tests showed that these also might have been random. However, 172 of these 3-combinations were located in four clusters each characterised by a defining genotype, and by inclusion of all patients having this genotype. Occurrence of three or more clusters of this type was not found once in 1000 permutation tests, indicating that at least some 3-combinations in the clusters may be related to BD. In addition, subgroups within the four clusters also shared the second genotypes, and some had the third genotype in the 3-combinations located in the same gene, suggesting that accumulation of several genotypes in a single gene may be important for the disease susceptibility in some cases.

We found only 5 nominally significant SNPs in common between the Scandinavian material and the WTCCC material (see Table S1), indicating heterogeneity between the two samples. A cluster with the same defining genotype as in one of the four clusters observed in our material could not be found in the WTCCC material, as none of the four defining SNPs were present among the 132 genotyped SNP or the 337 imputated SNPs in the WTCCC dataset. However, three significant clusters were found. Again three of more clusters were not seen once in 1000 permutation tests. Also nesting 3-combinations sharing the two first genotypes was present in the WTCCC dataset (p = 0.029). An overlap in the clusters observed in the two dataset were not see in the 3-combinations exclusively found in patients, but may be found in the much larger group of combinations common for control persons and patients.

An examination of individual patient data in both sample sets shows that most of the patients carry many of the 3-combinations in the clusters although none had exactly identical pattern of 3-combinations, raising the possibility that each patient has a unique genetic background for the disorder. The subgroups of 3-combinations with overlap in patients, sharing two genotypes in more than four 3-combinations, are interesting because different SNP as the third (sometimes even from the same gene (in some cases in close LD)) leads to an accumulation of several genotypes in small group of patients. Such an accumulation may be important for the disease susceptibility.

Askland et al. [12] found that although the data from two large independent GWAS [33], [35] both pointed to ion channel genes as important for BD, only a modest overlap between the two studies was found for the involved genes. The authors suggested that prominent genetic heterogeneity might explain this modest overlap [12]. The present analyses of the 3-combinations of SNP genotypes support the explanation that genetic heterogeneity is prominent in the genetic architecture of BD. This heterogeneity is illustrated by the many patient-specific 3-combinations of SNP genotypes, some of which may be important for BD. The selection of genes in this study is based on their relation to some aspects of signal transmission in the brain, so obviously any combination of genotypes might be related to this function. However, a more narrow relationship may be seen in the cluster defined by *KCNQ3* rs2469515 (Figure 4) together with *ANK3* rs12049756 and three different SNPs from *SCN2A* (rs12469667, rs3769949, and rs997508); as the proteins translated from these genes all are located in the node of ranvier [25]. Similarly, in the largest subgroup defined by *CACNG2* rs2179871 (Figure 3), and involving 13 combinations with *KCNN3* rs6426998 as the second SNP, many of the nine different genes involved as the third SNP are implicated in the architecture of the sodium channels in the node of ranvier (e.g. *SPTBN4*, *CNTNAP2*, *NFASC*, *SCN2A* and *ANK3*).

Our study indicates that BD may show extreme genetic heterogeneity at the population level. At the same time the many 3-combinations in each patient may support gene-gene interactions or epistasis important for BD. However, such interactions probably will involve genes not analysed in the present study. A more profound discussion of functional connections between the genes participating in a combination remain speculative and is preliminary until more genes related to signal transmission are analysed and combinations of more than three genotypes can be carried out. The pronounced genetic heterogeneity and the number of possible interactions on the individual level both suggest that the biology of BD may be very complex; but on the other hand, if the genotypes behind the heterogeneity are associated with a limited number of functions the degree of complexity may be decreased.

### Future direction

In this work we have looked at the combinations of three SNP genotypes that were observed in patients only. The next step is to look at the much larger number of combinations seen in both patients and controls. However, this may involve calculations of combinations with more than three SNP genotypes.

## Materials and Methods

### Patient sample

This study is based on two independent case-control samples from Norway and Denmark, included in the Scandinavian Collaboration of Psychiatric Etiology (SCOPE). The Danish sample consisted of 220 bipolar patients from the Copenhagen area, 162 bipolar patients in Jutland, and 1133 control participants. The sample from Norway included 222 controls and 225 bipolar patients. Thus, a total of 607 unrelated patients and 1355 unrelated healthy control participants were included. The Norwegian patients had been diagnosed according to the DSM-IV and the Danish patients according to ICD-10. The Norwegian and Danish healthy controls and cases have been described in more detail elsewhere [36], [37]. The Norwegian Scientific-Ethical Committees, the Norwegian Data Protection Agency, the Danish Scientific Committees, and the Danish Data Protection Agency approved the study. All patients gave written informed consent prior to inclusion in the project.

### Genes

The genes, shown in Table 1, were selected based on the following criteria: (1) literature relating corresponding functional proteins to various aspects of the action potential (with focus on the architecture of the sodium channels in the nodes of ranvier and CNS myelination) [25]–[29], [38]–[41]; (2) occurrence in loci associated with BD, as indicated by linkage analyses [30], [31]; (3) encoding of corresponding proteins that are targets for mood stabilizing drugs [29], [31], [32]; and (4) identification in previous studies showing an association with BD [26], [29], [31], [41]–[43]. Additional we used information from different internet sources (www.ncbi.nlm.nih.gov/Database/; www.ensembl.org/index.html; www.pubgene.org; www.ensembl.org/index.html; www.string.embl.de; www5.appliedbiosystems.com/tools/pathway/all_pathway_list.php; www.reactome.org).

We prioritized genes fulfilling more than one criterion, but realize that other genes fulfil the criteria and thus could have been included instead. The genotyping were planned in the autumn of 2007 and preformed in the spring 2008.

### SNP selection and genotyping

To cover most of the common variants with tagSNPs, we used a structured gene-wide approach, based on the HapMap CEU population. TagSNP selection was performed at the HapMap website using pair-wise tagging, with r^2^≥0.8 [44] (www.hapmap.org; HapMap Data Rel 22/phaseII Apr07) and minor allele frequency (MAF)≥0.05. Some SNPs (some with MAF<0.05) were selected if they resulted in a missense mutation or if they had been linked to BD. Not all selected tagSNP were genotyped, and exclusion was attributable to the following reasons: (1) a design score <0.4 for the Illumina Platform (n = 109); (2) failure during the genotyping analysis (n = 76); or (3) being discarded during quality control for several reasons [sample call rate <90%, more than three clusters seen in the result, and/or SNP not in Hardy-Weinberg equilibrium (HWE; p<0.001) (n = 94)]. This resulted in 803 SNP for analyses as described below.

Genomic DNA was extracted from whole blood. Most (796) SNPs were genotyped using the GoldenGate 1536plex assay (Illumina Inc.) on Illumina BeadStation 500GX at the SNP Technology Platform, Uppsala University, Sweden (www.genotyping.se), accredited by the Swedish accreditation agency SWEDAC, and approved according to a quality system based on the international SS-EN ISO/IEC 17025 standard. For the subset of SNPs used in this study, the reproducibility was 99.999% (there was one duplicate error in 70,098 duplicate genotype calls), and the average sample call rate per SNP assay was 99.6%. The four SNPs in *YWHAH* and the three SNPs in *SCN1B* were genotyped using TaqMan genotyping assays according to the manufacturer's instructions. For these, the reproducibility was 99.72%, and the average sample call rate per SNP assay was 98.4%.

### Statistics and data processing

The samples were tested for population stratification by calculating the gene-based overall fixation index F_ST_ using Arlequin Software [36], [37]. The statistical significance of single genotype distribution was assessed using the Chi-square or Fisher's exact test, whereas patterns of SNP combinations were assessed by permutation tests.

Combinations of genotypes were calculated using array-based mathematical methods [45], [46], where data are represented geometrically, hereby facilitating parallel processing, which was performed using programs from Genokey (www.genokey.com) and Dyalog (www.dyalog.com).

Each of the 1000 permutation tests (in our dataset) were performed as follows: 1) A permutation of the entire population (i.e. indicies 1 to 1962) is determined. The result is a new vector with all indices 1 to 1962 in random order. 2) 607 random “patients” are selected, and the remaining 1355 individuals are “controls”. 3) The cluster analysis on the 607 “patients” and 1355 “controls” is determined using exactly the same methods as previously on the biological samples. Likewise, permutation tests were performed in the WTCCC dataset with 1998 random “patients” and 1500 random “controls”.

### Imputation of WTCCC data

We obtained genotype data on the bipolar patients (n = 1998) and the UK blood service control group (n = 1500) from the WTCCC data [33]. We searched for SNPs genotyped in both samples and found 132. To get more SNPs in common for the two dataset, we performed imputation using Impute2 (mathgen.stats.ox.ac.uk/impute/impute_v2.html) [47], [48] of SNPs in the chromosome regions around our selected genes (with at minimum of 500 kb surrounding area). We used both the provided hapmap3 dataset and the 1000 genome project samples as reference samples resulting in “Ne” to be 15000. We used default setting with the following exception: we set “iter” to 40 and “k” to 100 to get better genotypes, we selected a call thresh hold to be 0.9, and set the result to include only the SNPs, that we have genotyped. Prior to the imputation we exclude SNPs from the WTCCC dataset, that WTCCC had excluded due to low genotyping quality. We only include imputated SNPs with a certain (more than 80% probability for a given genotype) genotype calling in more than 80% of the subjects.

## Supporting Information

Table S1 **Nominal significant SNPs in the present sample as well as in the WTCCC dataset.**
(DOC)
