# Supplementary material for: Combinations of SNPs Related to Signal Transduction in Bipolar Disorder
Source: PLoS One. 2011 Aug 29;6(8):e23812. doi: 10.1371/journal.pone.0023812 (PMC3163586; doi:10.1371/journal.pone.0023812)
Supplement: Table S1 — Nominal significant SNPs in the present sample as well as in the WTCCC dataset. (DOC) [file pone.0023812.s001.doc]

Table S1: Nominal significant SNPs in the present sample as well as in the WTCCC dataset.

|  | Significant SNP only in the Scandinavian sample | | Significant in both samples | Significant only in WTCCC dataset |
| --- | --- | --- | --- | --- |
| Gene | Not present in WTCCC | Present in WTCCC |  |  |
| *ANK3* | rs12049756  rs17230650 | rs1010556 (I) ^a^  rs10994322 (G) ^b^  rs11593565 (I)  rs1981251 (G) |  | rs10761482 (I)  rs10821717 (I)  **rs6479700 (I)** |
| ATP1A2 | rs11265329 |  |  |  |
| *ATP1A3* |  |  |  | rs2217342 (I)  rs4803520 (I)  rs8107107 (I) |
| AVPR1B | **rs33976516**  rs33990840 |  |  |  |
| BDNF |  | rs908867 (I) | rs7124442 (I) |  |
| *CACNG2* | **rs2179871** |  |  | rs2283996 (I)  rs9619621 (G) |
| CAMKK2 | rs11065502 |  |  |  |
| CNTN1 | rs11179132  rs11179168  rs17552679 |  |  |  |
| CNTN2 | rs11240351  rs16855045  rs16937  rs2229866  rs2229868  rs3767298  rs6656507 | rs1042831 (I) | rs4951162 (G) |  |
| CNTNAP2 | rs2159472  rs4431524 | rs10238991 (G)  rs1024676 (I)  rs10272638 (I)  rs2620460 (I) |  | rs10952747 (G)  rs1526147 (I)  rs1534709 (I)  rs1587048 (I)  rs2204412 (I)  rs4726913 (I)  rs6464757 (I)  rs6945513 (I) |
| ERBB4 | rs839523 | rs707284 (I) |  |  |
| IMPA2 |  | rs583965 (I) |  | rs613993 (I)  rs628419 (G)  rs636173 (G) |
| *KCNC1* | rs7110441 |  |  |  |
| KCNC2 |  | rs11180370 (I) |  | rs12321121 (I) |
| KCNN3 | **rs884664** | rs1218575 (I) |  | rs11264254 (I)  rs11578178 (I)  rs6699080 (I)  rs951241 (G) |
| *KCNQ2* | rs4809302 |  |  | rs3746367 (G) |
| KCNQ3 | rs2469515  **rs2469615** | rs16904608 (I)  rs2272679 (I)  rs2436133 (I)  rs2469520 (I)  rs7008160 (I) | rs17595945 (I) |  |
| MAG |  |  |  | rs12461927 (I) |
| MBP | rs12962017 | rs2282557 (G) |  |  |
| MCHR1 |  | rs133074 (G) |  |  |
| *MCTP2* | rs3784651 |  |  |  |
| MOG |  |  |  | rs3130253 (I) |
| NCAM1 | rs17115280  rs4646981  rs4646982  rs584427 |  |  |  |
| NFASC | rs2246662  rs3820336  rs7534993 | rs6593917 (G) |  | **rs12737855 (I)**  rs2841633 (I)  rs6659514 (I)  **rs7519658 (I)**  rs9194 |
| NRCAM |  | rs12538050 (I)  rs1269673 (I)  rs6975557 (G) |  | rs17155421 (I) |
| NRG1 |  | rs17731912 (I) | rs327329 (I) | rs2466051 (I)  rs2466094 (I)  rs3757930 (I) |
| P2RX7 | rs1718119  rs1718134  rs1718161  rs507085  rs7958311 | rs208296 (G)  rs2686369 (I)  rs6489794 (G) |  | rs2230912 (I) |
| PPP2R2C | rs10213410  rs4386675 | rs6838132 (I) |  | rs17721365 (I) |
| SCN2A | rs6738837 |  |  |  |
| SCN5A |  |  |  | rs6599230 (I) |
| SCN8A |  |  |  | rs1483000 (I)  rs303815 (I)  rs3900362 (I)  rs4761829 (I) |
| SPTBN4 | rs41334250  rs8107961  rs814501 | rs11672523 (I) |  | rs1165840 (I) |
| TNC |  |  |  | rs12347433 (I) |
| TNR | rs2021832 | rs2236883 (I) |  | rs9283389 (I) |
| TRPM2 | rs1618355 |  |  |  |
| YWHAH | rs1049583 | rs3761432 | rs2858753 (G) |  |

a) Imputed SNP in the WTCCC dataset. b) Genotyped SNP in the WTCCC dataset.

The defining SNP in a cluster is marked as **blue and bold**.
